# FPGA Implementation of Image Registration Using Accelerated CNN

**DOI:** 10.3390/s23146590

**Published:** 2023-07-21

**Authors:** Seda Guzel Aydin, Hasan Şakir Bilge

**Affiliations:** 1 Department of Electrical and Electronics Engineering, Bingol University, Bingol 12000, Turkey; 2 Biomedical Calibration and Research Center (BIYOKAM), Gazi University, Ankara 06560, Turkey; bilge@gazi.edu.tr

**Keywords:** accelerated CNN, field programmable gate array, image registration, ultrasound

## Abstract

Background: Accurate and fast image registration (IR) is critical during surgical interventions where the ultrasound (US) modality is used for image-guided intervention. Convolutional neural network (CNN)-based IR methods have resulted in applications that respond faster than traditional iterative IR methods. However, general-purpose processors are unable to operate at the maximum speed possible for real-time CNN algorithms. Due to its reconfigurable structure and low power consumption, the field programmable gate array (FPGA) has gained prominence for accelerating the inference phase of CNN applications. Methods: This study proposes an FPGA-based ultrasound IR CNN (FUIR-CNN) to regress three rigid registration parameters from image pairs. To speed up the estimation process, the proposed design makes use of fixed-point data and parallel operations carried out by unrolling and pipelining techniques. Experiments were performed on three US datasets in real time using the xc7z020, and the xcku5p was also used during implementation. Results: The FUIR-CNN produced results for the inference phase 139 times faster than the software-based network while retaining a negligible drop in regression performance of under 200 MHz clock frequency. Conclusions: Comprehensive experimental results demonstrate that the proposed end-to-end FPGA-based accelerated CNN achieves a negligible loss, a high speed for registration parameters, less power when compared to the CPU, and the potential for real-time medical imaging.

## 1. Introduction

Medical imaging systems play an important role in disease diagnosis, staging, treatment selection, planning, guidance, and follow-up [1,2,3]. Ultrasound (US) imaging is a diagnostic imaging modality that is widely used because of its advantages of being non-invasive, non-ionizing, and painless [1,2]. US imaging systems are also more portable and provide more real-time feedback than other imaging modalities, such as magnetic resonance imaging (MRI) and computed tomography (CT). However, in addition to these advantages, there are also certain disadvantages. One is the formation of low-resolution and low-contrast images [4]. This makes it difficult for specialists to make a diagnosis by looking at the images. Another disadvantage is that traditional US processes can only examine a narrow area of view [5]. Various techniques have been investigated to visualize larger areas and increase image resolution. IR is one of the most important of these methods. IR is the process of combining images side-by-side or on top of each other by determining the transformation matrix between two or more images taken from different angles, times, or modalities for the same region of interest [6]. The IR process is performed using classical and deep learning (DL)-based methods [7,8,9,10]. DL is widely recognized as a state-of-the-art technique for a broad range of applications, particularly in computer vision [11], natural language processing [12], bioinformatics [13], robotics and control [14], biomedical signal processing [15], and IR processes [16,17,18]. The classical method has the disadvantage of being an iterative process, which limits its applicability, particularly for real-time applications [19]. Most studies have shown that DL-based IR produces better results than traditional iterative methods. Therefore, the methods used in these studies focused on DL methods. The convolution operation performed in the convolution layer of the CNN approach, which is a DL method, has a structure that is suitable for parallel operation. By revealing the parallel processing capability of the CNN structure, operations can be performed faster; this represents a great improvement, especially for real-time applications where speed is important [20,21]. However, this parallelism cannot be fully revealed using general-purpose processors suitable for sequential processing. Therefore, although studies using CNN methods are faster than classical iterative methods, the maximum speed that can be reached has not been fully achieved because the parallelism feature of CNN is not fully utilized when using general-purpose processors. In addition, because convolution operations result in intensive operations on the platforms on which they are applied, dedicated hardware such as graphics processing units (GPUs) and FPGAs would be more efficient for implementing CNNs [22]. CNN algorithms have been developed and accelerated on GPU platforms that have a high memory bandwidth and significant parallel computing capability. However, research on GPUs has revealed that they typically consume more power than FPGAs, making them inefficient and difficult to use in battery-powered devices [23]. As a result of their high performance, energy efficiency, and reconfigurability, FPGA-based implementations have attracted considerable interest [24,25,26,27]. Studies have demonstrated that FPGAs are faster than CPUs and more energy-efficient than GPUs. Thus, the focus of the studies has changed to the application of DL techniques to FPGAs.

Medical IR applications are performed using classical iterative methods and the recently developed DL methods which provide the best results, especially in computer vision fields. The researchers in [28] used an unsupervised CNN for IR of affine and deformable cardiac cine MRI and chest CT images. Their study consisted of two stages. As a first step, they created a CNN model for affine IR, which takes a moving and fixed image from two parallel branches, processes them simultaneously, and combines them towards the later part of the network. The output of the first model consists of 12 affine parameters. The second structure was created for deformable IR. This structure, which uses a fixed-moving image pair as its input, creates a B-spline 3D displacement vector as the output. They used different metrics to evaluate registration performance. Examples of these metrics include Dice (range from 0 to 1, 1 representing a perfect match between registered images), the Hausdorff distance (HD, low is significant), and the average symmetric surface distance (ASD, low is significant). They implemented the classical methods using SimpleElastix. They have designed many applications in their work. The values they obtained for one application they designed are as follows. In their cardiac MRI registration comparison study, Dice 0.86 ± 0.18, HD 9.76 ± 4.78, and ASD 1.14 ± 1.4 were calculated as a result of the study performed with classical methods. In the DL-based method, Dice 0.87 ± 0.18, HD 9.47 ± 5.26, and ASD 0.98 ± 1.12 were calculated. The runtime was calculated as 13.49 s and 1.71 s, respectively, for the classical method and the DL-based method on the CPU. Although both methods produce the same results according to the evaluation metrics, the DL-based method produces better results than the classical method in terms of speed. Recently, the authors of [29] presented a method in which, instead of training a large network at once, small-network training was performed, and the network was expanded gradually to estimate the displacement for complex deformation fields. The authors stated that their proposed method provided better results in estimating a large displacement area than using the full network all at once. They used a supervised learning method as the training model for CNN. Lung CT IR was performed as the data were processed. They used the target registration error (TRE in millimeters, lower means better results) as the assessment metric. The TRE was calculated as 3.39 ± 3.48 mm for the classical method and 2.37 ± 1.77 mm for the DL-based method. They made the runtime comparison with studies using the classical method in the literature. Despite two studies with a runtime of 7.97 min and 58 min using classical methods, the runtime of the proposed DL-based application was 0.55 s. Furthermore, in [30], a CNN and spatial transformer-based model were proposed to register US-to-CT images for cardiac arrhythmias and guidance therapy. They stated that their work on the non-rigid transformation model produced much faster results than the classical iterative methods. They implemented the classical methods using SimpleElastix. They used Dice, HD, and runtime as comparison parameters. According to these parameters, the classical method was calculated as 0.7, 1.7 mm, and 65 s, and the DL-based method was calculated as 0.8, 1.2 mm, and 0.7 s for Dice, HD, and runtime, respectively. Similarly, Ref. [31] proposed an unsupervised DL-based rigid US IR. Their DL-based model estimated the values of rotation, dx, and dy translation, which are three rigid transformation parameters, as outputs. They used the mean square error (MSE) as the evaluation parameter. While an MSE of 1.7 × 10^3^ was calculated for the classical method using SimpleElastix, a value of 1.59 × 10^3^ was calculated for the DL-based method. In addition, classical methods for 64 × 64 input images produced a response in 6 s; the method they proposed produced a response in 0.1 s on a GPU. According to their performance analysis on the US dataset, the method they proposed provided more accurate results in a shorter time than conventional methods. As these studies in the literature show, DL-based methods outperform classical methods in terms of speed, although they show the same registration performance and sometimes an even better performance than classical methods.

Recent studies have shown that the use of DL-based methods for image and signal processing in FPGAs has increased. The researchers in [32] proposed an FPGA-based acceleration method for parallel MRI reconstruction. As they noted, the computational requirements of MRI methods have increased to the point where using general-purpose computers for reconstruction would be impractically time consuming for real-time MRI. Therefore, they used an FPGA to perform hardware-based processing and shorten the reconstruction time. Their results showed that the proposed design outperformed its CPU counterparts while maintaining reconstruction quality, with a maximum speed gain of up to 298x.Similarly, in [33], the researchers proposed an FPGA-based accelerator for optical flow estimation. They used the line buffer technique to use the data. When compared to current optical flow accelerators, they claimed that extensive experimental results show that their proposed FPGA-based design achieves the highest estimation accuracy and real-time performance. In addition, in [34], fixed-point (FXP) implementation for hardware was used to assure energy-efficiency requirements for computing platforms. As a result of their work, the authors proposed a fixed-point implementation and used 32% fewer resources than the 32-bit floating-point implementation with negligible accuracy loss. Researchers in [35] proposed a two-design structure for Lenet-5-based traffic sign recognition. In their studies, they performed Lenet-5 on an FPGA using a single computation engine and streaming design models. In the final layers of Lenet-5, the fully connected layers were replaced with 1 × 1 convolution layers to save hardware costs and reduce the design complexity. According to them, the Lenet-5 application that they implemented on the FPGA produced results significantly more quickly than the application that they had previously implemented on the ARM processor. In [36], an FPGA-based CNN implementation was considered for object detection in optical remote sensing. The authors accelerated their designs by optimizing data storage and parallel processing. In their study, power consumption, frequency, throughput, and accuracy metrics were used to compare the CPU, FPGA, and GPU platforms. It was stated that the GPU with 5279.4 GOP/s obtained the best result for the throughput metric, and the FPGA platform attained the best result with a power consumption of 5.96 W, whereas the accuracy metric remained the same for all three platforms. In contrast to the aforementioned models, the authors of [37] investigated the feasibility of implementing a 3D CNN on the VC709 FPGA. They conducted a study to implement the Inception-based 3D CNN model on an FPGA and to investigate its implementation optimization parameters. The 70-layer model used by their 3D CNN is designed to predict action from video. First, they used a pre-trained model’s feature maps and weights to reduce network complexity with minimal impact on accuracy. Then, to increase memory bandwidth while decreasing the number of DRAM accesses, a data tiling technique is proposed.

In this study, the IR process was performed using an FPGA-based accelerated CNN. The aim of this study was to achieve quick and highly accurate US-to-US monomodal IR using a CNN via FPGA-based acceleration methods. In this study, convolution, pooling, and fully connected layers, which are the basic layers that make up the CNN structure, were created one by one on the FPGA. For the estimation of the rigid registration parameters, the network was trained on the software. At the end of the training in the software, the number of layers and widths and the filter sizes to be used were determined. According to these determined hyperparameters, the layers formed on the hardware are connected. The number of these basic layers can be increased in the design of larger and more complex networks without exceeding the amount of hardware resources to be used. Along with its use in the medical field, the image registration process is also utilized in numerous other fields, such as the creation of panoramic images [38], the registration of satellite images [39], remote-control applications [40,41], etc. Additionally, the CNN model is utilized in a variety of applications. After determining the parameters and hyperparameters of the network trained for various applications on the software, it can be implemented on the hardware by connecting the fundamental layers designed in this study. This will show the broader applicability and potential impact of the proposed FPGA-based accelerated CNN.

The contributions and novelties of this study are as follows:

- The software-based regressor model, whose parameters and hyperparameters were determined by training on the CPU, was successfully implemented on an FPGA for the inference phase.
- Successful rigid US-to-US monomodal IR was achieved using a simple FUIR-CNN. A fully connected layer was used only in the output layer to enable real-time applications by reducing the design complexity and minimizing the power consumption.
- An approach to implementing CNN via a streaming process is proposed, with the idea being that the blocks can be built with pipelining enabled on their interfaces and executed concurrently, leading to high processing parallelism.
- The three rigid transformation parameters obtained as the outputs of the CNN regressor network were compared using different metrics for the software and the proposed FUIR-CNN at the inference stage.
- In a hardware-based study, the effects of using different number representation methods to accelerate the network on hardware resource usage, latency, and regression performance were investigated. As a result of this study, it was decided to use a 20-bit FXP, whose regression performance was close to that of the software-based model, but with a low resource usage and latency.
- Parallelism between CNN layers was achieved using a dataflow optimization method. End-to-end accelerated CNN layers work concurrently. The pipeline and unroll techniques were used on the loops to parallelize the operations within the layers. FIFOs were used between layers to reduce the amount of data transmission and memory access.
- The proposed FUIR-CNN was validated by three US datasets. It produced results for the inference phase 139 times faster than the software-based network while retaining a negligible drop in regression performance.

The remaining sections of this study are arranged as follows. The Section 2 introduces the software approach for IR and FPGA-based accelerating techniques that include the parallelism methods used in the CNN and the number display formats used for the representation of numbers. In addition, the datasets used in this study and the metrics used to measure the performance of the proposed method are introduced. The Section 3 presents software- and hardware-based (proposed) implementations. Finally, in the Section 4, the results are discussed, the limitations of the study are mentioned, and future studies are planned.

## 2. Materials and Methods

### 2.1. Rigid Image Registration

IR is the process of placing two or more images on the same coordinate system to align or overlap them [16]. Different images of the same region may have been obtained by different imaging modalities, at different times, or from different angles. The IR process is based on finding the transformation matrix between two or more images. As demonstrated in Equation (1), the IR problem can be viewed as an optimization problem. I_M_ and I_F_ denote the moving image, and fixed image, respectively. T is a transform function that aligns IF and IM. T(I_M_) is the warped image created from I_M_ using the transformation T. The function S() evaluates the similarity between I_F_ and T(I_M_). As a result, the goal of IR is to find the T that maximizes image similarity while taking cost into account.

(1) $$\hat{T}={\mathrm{argmax}}_{T}S(I_{F},T(I_{M}))$$

In this study, the rigid transformation method is used as the transformation method indicated by T. Rotation (rot) and translation (dx, dy) are the three degrees of freedom (DOF) used to describe rigid registration. Homogeneous coordinates are used to represent rigid transformations. Accordingly, the 2D vector (x, y) can be represented as a 3D vector (x, y, 1). Using this method, the translation can be converted into a matrix multiplication operation. The rigid transformation is defined in Equation (2), where v represents the vector on which the operation is performed, T(v) represents the vector formed after the rigid transformation, R represents the rotation, and d represents the translation. A homogeneous representation of the translation and rotation matrix is given by Equations (3) and (4), respectively. Each pixel in the (x, y) coordinates in the original image was moved along a certain offset distance (dx, dy) and placed in its new position in the (x′, y′) coordinates.

(2) $$T\left(v\right)=\mathrm{Rv}+d$$

(3) $$\left[\begin{array}{c} x’\\ y’\\ 1 \end{array}\right]=\left[\begin{array}{ccc} 1 & 0 & \mathrm{dx}\\ 0 & 1 & \mathrm{dy}\\ 0 & 0 & 1 \end{array}\right]\left[\begin{array}{c} x\\ y\\ 1 \end{array}\right]$$

(4) $$\left[\begin{array}{c} x’\\ y’\\ 1 \end{array}\right]=\left[\begin{array}{ccc} \cos\theta & -\sin\theta & 0\\ \sin\theta & \cos\theta & 0\\ 0 & 0 & 1 \end{array}\right]\left[\begin{array}{c} x\\ y\\ 1 \end{array}\right]$$

**Figure 1:**
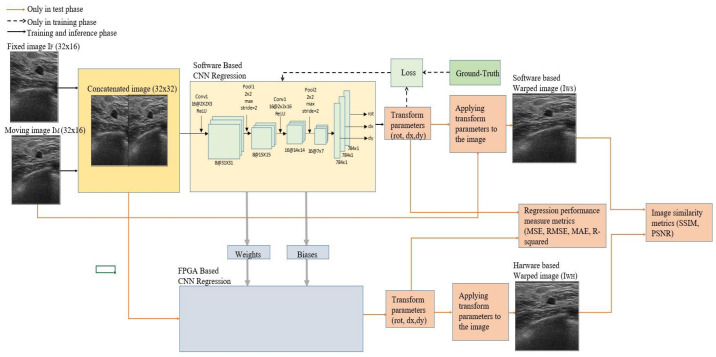
The overall structure of the study.

The proposed overall structure of the study is illustrated in Figure 1. The study consists of two stages, software-based and hardware-based, as shown in Figure 1. The designed network has a single input and multi-output structure. The regression models take two 32 × 16 US B-mode image pairs (I_F_ and I_M_) as inputs as 32 × 32 concatenated images. The CNN model consists of two convolution layers and two pooling layers. The fully connected structure is used only in the output layers. In the software-based study, the CNN network was trained, and the weights and bias parameters were recorded for use in the hardware-based CNN after the training was completed.

A supervised learning method was used as the learning method for the CNN. As a result of a supervised model being used, ground-truth values were required for the training phase of a software-based study. The ground-truth values used during training were created synthetically. During training, the network calculates the loss value by comparing the ground-truth values with the predicted values. Ground values and loss calculation processes were used only in the training phase. There are three independent output layers. The boxes indicated by the transform parameters are the three DOF parameters. The outputs of the networks are rot, dx, and dy, which are used to calculate the mean absolute error (MAE), mean squared error (MSE), root MSE (RMSE), and R-squared for the software- and hardware-based networks’ prediction performance. The CNN was trained on three US datasets with a synthetic random transformation, which was utilized for generating the image pairs for the desired application. In addition, the I_WS_ and I_WH_ images were obtained by applying the transformation matrix predicted by the software- and hardware-based CNN to the moving image I_M_. The similarity between the I_WS_ and I_WH_ images was calculated with the structural similarity (SSIM) and peak signal-to-noise ratio (PSNR). Comparisons made in this study were made between the results produced by the software and hardware-based CNN models.

### 2.2. FPGA-Based CNN

FPGAs are devices with semiconductor technology that are widely used today, particularly in the industrial, military, and medical sectors. The FPGA is a hardware circuit that allows the user to program it to perform one or more logical operations. Programmable logic gates typically include memory and other components. The capability to perform parallel operations is the most significant and distinctive feature. Hardware description languages, such as VHDL or Verilog, are generally used for modeling the FPGA platform [42]. However, these languages are difficult and time-consuming to learn compared with other programming languages. As an alternative, high level synthesis (HLS) technology, which has been increasingly used for FPGA programming, has recently emerged. Vivado HLS allows users to easily build complex FPGA-based algorithms using C/C++ code. C/C++ codes are converted to VHDL or Verilog codes with HLS and then converted into IP packets that can be embedded in the FPGA. The created algorithms can be verified both functionally for C-level languages and at the register-transfer level (RTL) at the hardware level. In this study, the FPGA-based FUIR-CNN structure was verified at the C level in HLS using C++ codes, and the synthesized circuits were verified with C/RTL co-simulation. The synthesized circuits were turned into an IP and manufactured ready to be embedded in the FPGA.

### 2.3. Hardware Accelerator

#### 2.3.1. Data Type for Efficient Hardware Implementation

The number representing format and length to be used in the design, the parallelism methods to be used in the calculation units, and the data-storage method are the three important issues that should be considered for accelerating the CNN. The formats of the data types used in hardware-based system designs affect the performance of the system in several ways, such as resource usage, accuracy, and latency.

**Figure 2:**
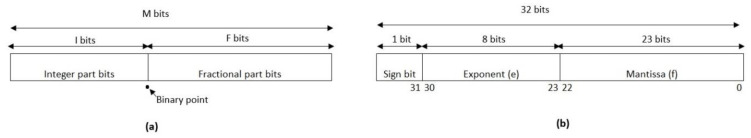
Number format: (**a**) fixed point, (**b**) 32-bit single precision floating point number structure.

In hardware platforms, there are two basic approaches to represent numbers with fractional components: fixed-point and floating-point (FP) representations, as shown in Figure 2. In Figure 2a, the binary point divides a number’s integer and fractional parts for fixed-point representation. M represents the total number of bits used to represent the number. I and F, respectively, represent the bit numbers above and below the binary point. The bit width for the integer part and the fraction part can be chosen as desired according to precision.

Floating-point numbers are preferred because they have a wide range of number representations. According to the IEEE 754 standard [43], the numbers are represented as 32 bits in binary format. This notation has three basic components: the mantissa, the exponent, and the sign bit. The biggest advantage of using floating-point numbers is that their computational precision is very high. Thus, the accuracy of the results produced by the network is also high. However, in hardware-based studies, this high-precision number format causes significant resource consumption. Consuming too many resources also results in increased power usage. For hardware, it is very important to balance the performance of the network, the number of resources consumed, and the power consumed. Using the correct data representation type provides a better performance for a hardware implementation that is nearly as accurate while running faster and using fewer hardware resources.

The Vivado HLS tool supports the C/C++ float and arbitrary precision data-types by including libraries and header files suitable for these number notation methods [44]. Floating point number representation can be used by adding a float to variable definitions and adding the required library. Likewise, if arbitrary precision number representation is desired, the number of bits to be used for number representation should be determined first, and this new type should be named [44]. Vivado HLS automatically performs mathematical operations with these number representation techniques.

#### 2.3.2. Parallelism for FPGA-Based CNN

**Figure 3:**
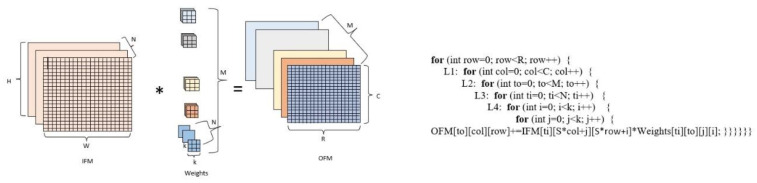
Convolution operation for a multi-channel image and pseudocode of the convolution operation.

The convolution, pooling, and output layers are created by setting nested loops and arrays, which are the building blocks of the HLS-based CNN implementation. Loops in the process commonly consume a significant portion of the execution time; therefore, it is important to consider time-critical loops. Figure 3 represents the convolution process for a multi-channel image and the pseudocode of the convolution operation. The convolution process consists of six nested loops. N and M represent the channel numbers of input feature maps (IFM) and output feature maps (OFM), respectively. C and R represent the height and width of OFM; k represents the size of the kernels. Pool operations and fully connected layer operations are also performed using for loops. Therefore, the fundamental units of FPGA-based CNN acceleration processes are nested for loops.

##### Pipeline

**Figure 4:**
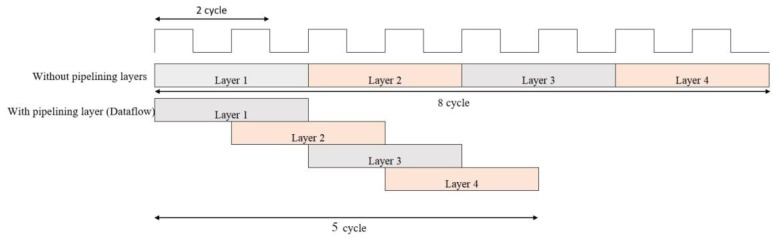
Pipeline optimization technique between the layers.

Different parallel operations can be performed for the FPGA-based CNN architecture. One of these methods is the interlayer parallelism method. In the CNN inference phase, all layers are dependent on each other. That is, layer (l) takes the feature maps (FMs), which are the outputs of the previous layer (l-1), as input and starts the process. Therefore, the layers cannot work completely in parallel; instead, partial parallelism is achieved using the interlayer pipeline method, as illustrated in Figure 4. The dataflow applied design completes all operations in five cycles, while the design without dataflow takes eight cycles to complete layer operations.

##### Unroll

**Figure 5:**
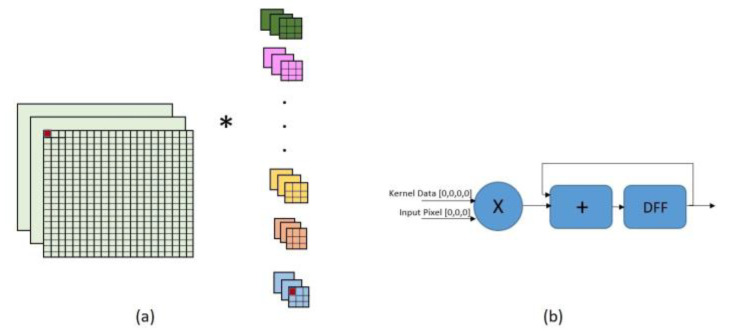
The effect of the rolling nested loops on (**a**) input tensors and kernels and (**b**) hardware resource usage.

One of the most important optimization methods for the loop is the unroll technique. Convolution operations are performed concurrently using the unroll technique, which optimizes the CNN execution time. Rolling loops create hardware resources that are used by each iteration. The approximate effect of rolling loops on hardware resource transfer is shown in Figure 5.

**Figure 6:**
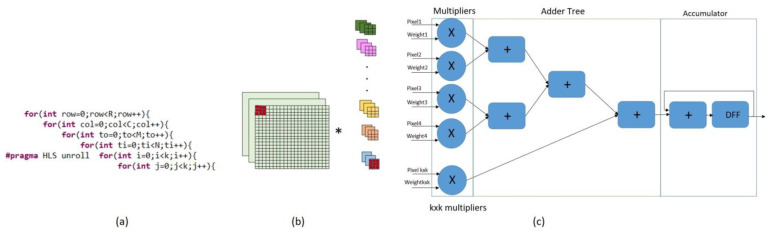
The implementation of the unroll pragma (**a**) at the kernel level on the pseudocode of the convolution operation, (**b**) on the input tensors and kernels, and (**c**) on hardware resource usage.

When the unroll technique is applied, hardware resources are assigned to each process, allowing the processes to be performed in parallel. Figure 6 shows (a) the implementation of the unroll pragma for the kernel level on the pseudocode of the convolution operation, (b) the demonstration of unroll optimization on input tensors and kernels, and (c) the impact on hardware resource usage.

The convolution operation can be executed in parallel in different ways using the unroll technique. Unrolling each of the nested loops shown in Figure 3 corresponds to a different parallelism method. This also causes weight and IFM data to be shared in different ways [45].

**Figure 7:**
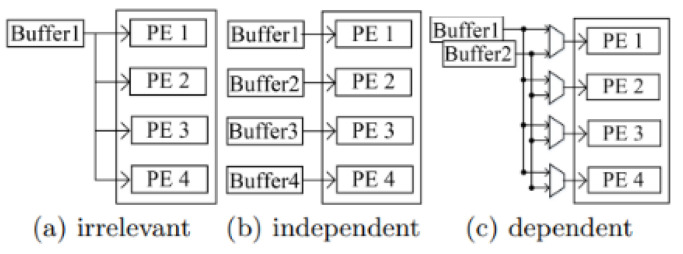
Hardware implementation of different data-sharing relationships [45].

There are three datasets processed in layers. These are IFM and weights, which come as input data to the layers, and OFM, which is the data group that comes out of the layers. IFM, weights, and OFM data sharing can happen in three different ways as a result of unrolling different loops [45]. They are categorized as irrelevant, independent, and dependent, as shown in Figure 7. PE represents the units of calculation. In the irrelevant method, the same data are sent to each computation unit. In the independent method, the data sent to each computation unit are different.

##### Array Partitioning and Data Storage Manner

The input image data, bias, and weight parameters used in the CNN design and the output data formed at the end of each layer should be stored. BRAM resources can be used to store large amounts of data on an FPGA. However, BRAM units allow a maximum of two data points to be output simultaneously. This prevents the attainment of the maximum performance that parallel processing units can achieve. The array-partitioning method is used as the memory optimization method. In the array-partitioning method, BRAM units are divided into smaller BRAM units, or registers, allowing more than one datum to feed the units in parallel at the same time. Another method used for data storage is the buffering technique. Using buffer units, only sufficient data to be processed at a time are stored. By storing these data in registers, they can feed processing units in parallel. Another method to store data is to use FIFO storage units instead of BRAM resources. FIFO storage units enable data storage without the need to address the information required in BRAMs. Using FIFOs after each layer for designs in which the layers are processed with the pipeline method will ensure that the information going to the next layer is transmitted quickly and that the outgoing data are freed up to make room for the next data.

### 2.4. Proposed CNN Accelerator for US Image Registration

**Figure 8:**
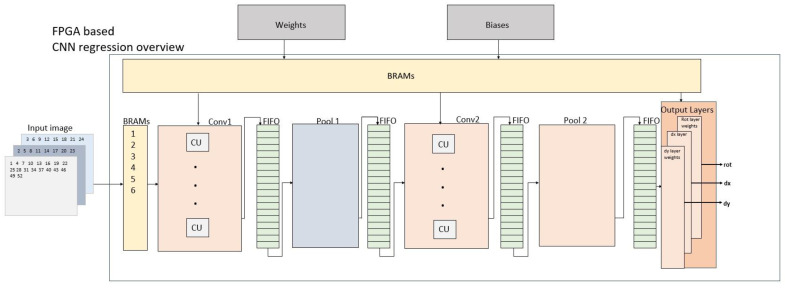
Proposed FUIR-CNN overall structure.

**Figure 9:**
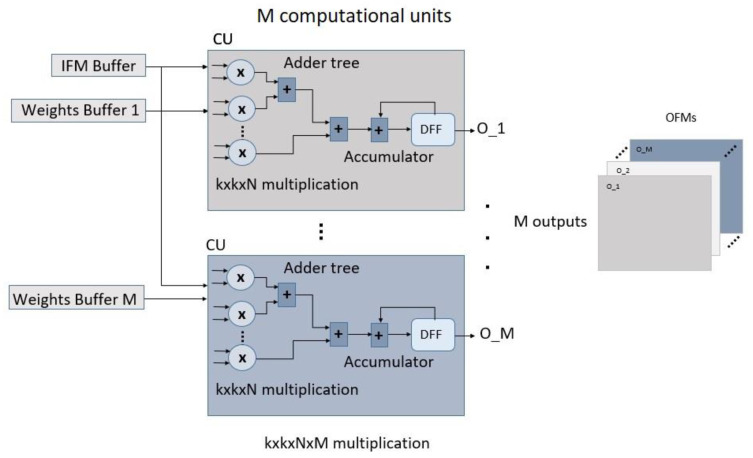
Proposed computational units.

There are two design approaches for CNN applications based on FPGAs: streaming and single computation engines [46]. In the single computation unit structure, a uniform unit is designed, and the layers are sequentially implemented within this unit. In a streaming structure, each layer is independently designed and implemented. The proposed CNN is illustrated in Figure 8. Streaming was used as the design method for FUIR-CNN. In streaming design, each layer of the CNN structure is designed and implemented separately. Dataflow optimization is used between the layers. OFMs formed after each layer are stored in FIFO units. This is more advantageous than using BRAMs to store OFMs, as the values stored in FIFO units are immediately transferred to the next layer. The weight and bias values obtained from the software-based trained structure were used in the implementation of FUIR-CNN. BRAMs were used to store the bias and weights of the Conv1, Conv2, and FC layers. The L1 loop, as shown in Figure 3, was pipelined so that the operations could be performed concurrently. Then, the L2, L3, and L4 loops were unrolled to improve pipelining and decrease latency. The effect of unrolling these loops is shown in Figure 9. The computation unit (CU) represents the unit in which the convolution operation is performed. In the CU, a three-dimensional multiply-accumulator (MAC) is performed. FPGAs use DSPs to perform MAC operations. One CU corresponds to the unit that provides the calculation of one pixel of OFM. The multiplication and accumulator numbers used in CU units vary according to the number of filter sizes and input–output channels. In every cycle, k × k × N × M parallel multiplications are performed. There are 8 and 16 CU units for the Conv1 and Conv2 layers, respectively. There are (2 × 2 × 3 × 8) 96 and (2 × 2 × 8 × 16) 512 parallel multiplications in the Conv1 and Conv2 layers, respectively.

The software-based CNN model consists of three independent output layers. In the FUIR-CNN implementation, we combined these three separate layers into a single layer that gives three outputs of 724 × 3. Thus, the three outputs occur sequentially, not simultaneously.

### 2.5. Datasets

The CNN network was trained using three different US datasets. However, publicly available datasets are insufficient for network training. Thus, data augmentation processes were used to increase the amount and variety of data.

#### 2.5.1. BUSI

The BUSI dataset [47] contains US B-mode images grouped into three classes: normal, benign, and malignant. These data, collected at baseline and consisting of breast US images from 600 female patients aged 25–75 years, were collected in 2018. This dataset included 780 US images with an average image size of 500 × 500 pixels in the PNG image format. The images in this database are prepared for classification, detection, and segmentation. There are also ground-truth files that serve this purpose. However, in this study, this ground truth was not used because image registration was performed. Only the original images were used.

#### 2.5.2. Breast Ultrasound Lesions Dataset (Dataset B)

Dataset B [48] has two classes, malignant and benign, and consists of 163 US B-scan images. The objective of this dataset is to provide images and ground truth for the automatic interpretation and analysis of breast ultrasound images. This dataset, collected from a different partition, has a mean image size of 760 × 570 pixels and was collected in 2012 with a Siemens ACUSON Sequoia C512 system 17L5 HD linear array transducer (8.5 MHz). Of the 163 US breast images, 53 and 110 were of the malignant and benign classes, respectively.

#### 2.5.3. B-Mode Common Carotid Artery (CCA) Ultrasound Image Database

This database [49] contains 84 longitudinal B-mode ultrasound images of the CCA from ten volunteers. Approximately eight US images were scanned from each volunteer with different gain compensation curves, depths, gain times, and linear array transducers. Images have a resolution of approximately 390 × 330 pixels. The exact resolution of an ultrasound scanner depends on its configuration. Two distinct linear array transducers with different frequencies (10 MHz and 14 MHz) were utilized. Images are in JPEG format.

### 2.6. Data Augmentation

The number of medical US images obtained from the specified datasets was low; thus, the number of images to be used for training was increased through data augmentation processes. This could partly address the problem of a small number of US images. Thus, minimizing overfitting and strengthening the generalization capability of the DL network was possible. Publicly available datasets usually consist of data acquired for classification or segmentation. In this study, while data augmentation was performed on the images used for IR processing, the ground-truth values, including the inverse transformation matrix, were recorded. For the data augmentation process, rotation, scaling, and translation processes were applied, which represent one of the affine transformation methods, considering the structure of the images. Affine transformation is a linear mapping technique that keeps planes, lines, and points intact [50].

**Figure 10:**
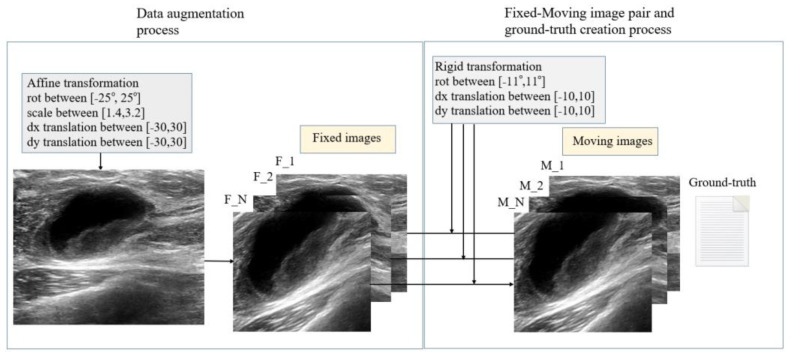
Data augmentation and dataset preparation for the train-test phase.

In Figure 10, three processes are shown: data augmentation, the creation of fixed-moving image pairs to be used in the training-test stages using the increased new data, and the ground-truth generation processes in which the transformation matrices between these image pairs were recorded. By applying a random transformation that includes scaling by a factor in the range of [1.4, 3.2], rotating by an angle in the range of [−25°, 25°], and translating horizontally and vertically by a distance in the range of [−30, 30] pixels to the three above-mentioned US datasets, the number of images was increased to 10,230. Thus, the number and variety of images to be used in the study were increased, and then all the images were converted to 32 × 16 × 3 dimensions in JPEG format. Other than these operations, no preprocessing was conducted. Fixed and moving image pairs and the transformation matrix between them were needed for the registration process. Therefore, as a second step, fixed-moving image pairs and a ground-truth-containing transformation matrix between them were created. The rigid transformation to be applied in this study consisted of three parameters called degrees of freedom (DOF). These three DOF parameters are rotation (rot), translation in the x-axis (dx), and translation in the y-axis (dy). Moving images were created by applying rotation and dx, dy translation processes to fixed images. Rotations in the range of [−11°, 11°] clockwise (negative rotation value) and counterclockwise (positive rotation value), dx translation in the range of [−10, 10] pixels in the x-axis, and dy translation in the range of [−10, 10] pixels in the y-axis were applied to the fixed images. The ground-truth values of the resulting fixed-moving image pairs, including the inverse transformation matrix, were recorded.

### 2.7. Evaluation Metrics

The registration performance was calculated for the output of the networks using MAE, MSE, RMSE, and R-squared. Moreover, the SSIM and PSNR image similarity metrics were used to evaluate the registered image pairs. Equation (5) demonstrates that the MSE measures the mean of the squares of errors, that is, the mean squared difference between the estimated values and the actual value. With MAE, as shown in Equation (6), the average size of the prediction error is calculated. The RMSE, shown in Equation (7), is the square root of the MSE. It is more frequently used than MSE because the MSE value can occasionally be too high for easy comparison. As a result, the MSE is computed using the square of the error, making the interpretation easier.

(5) $$\mathrm{MSE}=\frac{1}{N}\overset{N}{\underset{i=1}{\sum }}{\left(y_{i}-\hat{y_{i}}\right)}^{2}$$

(6) $$\mathrm{MAE}=\frac{1}{N}\sum_{i=1}^{N}\left|\left(y_{i}-\hat{y_{i}}\right)\right|$$

(7) $$\mathrm{RMSE}=\sqrt{\frac{1}{N}\overset{N}{\underset{i=1}{\sum }}{\left(y_{i}-\hat{y_{i}}\right)}^{2}}$$

(8) $$R-\mathrm{squared}=1-\frac{\sum_{i=1}^{N}{\left(y_{i}-\hat{y_{i}}\right)}^{2}}{\sum_{i=1}^{N}{\left(y_{i}-\bar{y_{i}}\right)}^{2}}$$

(9) $$\mathrm{PSNR}=10\log_{10}\frac{{\mathrm{MAX}ı}^{2}}{\mathrm{MSE}}$$

(10) $$\mathrm{SSIM}\;\left(I_{F,}I_{W}\right)={\left[I\left(I_{F,}I_{W}\right)\right]}^{\alpha }.{\left[c\left(I_{F,}I_{W}\right)\right]}^{\beta }.{\left[s\left(I_{F,}I_{W}\right)\right]}^{\gamma }$$

N, $y_{i}$, $\hat{y_{i}}$, and $\bar{y_{i}}$ represent the number of samples, actual values, predicted values, and mean of the y value in the equations, respectively. R-squared evaluates how well the regression line fits the original data, as shown in Equation (8). The R-squared values range from 0 to 1. A high R-squared value indicates a good regression model fit. PSNR is the similarity between two images and is used as a quality measure. It is the ratio of the maximum pixel value to the noise that affects the quality of the pixels and is usually expressed in decibels on a logarithmic scale. The higher the PSNR value, as shown in Equation (9), the better the quality of the output image. MAX_ı_ is the maximum valid value for the image pixels. SSIM, as illustrated in Equation (10), measures the similarity of images in three parts: luminance (l), contrast (c), and structure (s). The variables α, β, and γ in the function represent the effect ratios of the comparisons. SSIM takes values between 0 and 1. A value close to 1 means that the similarity is high.

The FPGA-based CNN’s performance is calculated using latency, throughput, hardware resources, and power consumption. Latency is the number of clock cycles required for the function to calculate all the output values. Hardware resources are defined here as the hardware resources required to implement the design based on the resources available in the FPGA, including LUTs, registers, BRAM, digital signal processing (DSP48s), and FFs. Large amounts of data are stored on FPGAs using BRAMs. The number of BRAMs used depends on the input image, the number of weight and bias parameters, and the length of the bits used to represent the numbers. Different arithmetic operations, including multiply-accumulators and multiply-adders, are usually implemented in DSP units. The availability of DSP resources in an FPGA is a factor in parallel convolution operations. As the number of parallel processes increases, the number of DSPs used also increases. Throughput is a parameter used to measure the FPGA’s performance. The capability of a function call to complete a clock cycle is referred to as throughput. This is usually calculated in giga operations per second (GOP/s).

## 3. Implementation and Results

This study was conducted in two stages. In the first step, the network was trained using a CNN architecture for the medical IR problem. In the second step, the determined CNN structure was implemented on the FPGA. The hardware-based CNN structure was created using the hyperparameters that formed the model, as well as the weight and bias parameters recorded after the training was completed.

### 3.1. Software

The training CNN model was implemented in a Python environment using Keras with a TensorFlow backend on an Intel(R) Core(TM) i7-10750H 2.60 GHz CPU and 16.0 GB RAM. The Adam optimizer was employed, and the learning rate (LR) was 0.00001. MSE was used as the loss function. In total, 20,460 US B-mode images were used in this study. The images were then divided into 10,230 fixed–moving pairs. In total, 8184 image pairs were used for training, 1842 image pairs were used for validation, and 204 image pairs were used for testing. The images used for testing were not used during network training.

In addition, the size of the images was reduced to 32 × 16 × 3 pixels. The pairs of images feeding the input to the network were also concatenated. Thus, the image size created for the network input was 32 × 32 × 3 pixels. The batch size was 50, and the number of epochs was 15,000. The network provided three output parameters: rotation, translation along the x-axis, and translation along the y-axis, with rotation in the range of [−11°, 11°], dx translation in the range of [−10, 10] pixels on the x-axis, and dy translation in the range of [−10, 10] pixels on the y-axis. The images given to the network were normalized between 0 and 1. Ground-truth values were normalized between −1 and 1. The hyperparameters of the model, determined after various trials, are listed in Table 1. To prevent overfitting and reduce the number of parameters so that the model can be used in hardware designs, a structure with a low number of layers was preferred considering the estimation performance in the studies. The determined structure consisted of two convolution layers, two max-pooling layers, a dropout with 0.5, and three output layers (rot, dx, and dy). The rectified linear unit (ReLU) was used as the activation function for the convolution layers. The activation function was not used in the output layers, and linear outputs were used. The performance of the network in estimating the three rigid transformation parameters was calculated using the MSE as 0.0551, 0.2802, and 0.1374, respectively, for 204 fixed-moving test image pairs. The training and validation losses for the three outputs of the network are shown in Figure 10.

**Figure 11:**
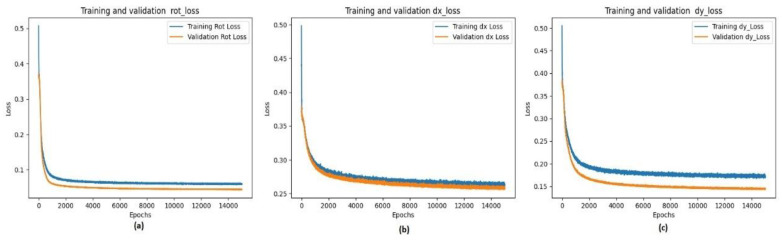
Loss graphs for train and validation sets of the three outputs: (**a**) rot, (**b**) dx, and (**c**) dy.

As shown in Figure 11, the validation loss rate is lower than the training loss rate. This is because of the use of the dropout layer. The dropout layer was used during the training phase but not in the verification and testing phases. This caused the training loss value to be higher than the validation loss value.

### 3.2. Hardware

The weight and bias parameters of the CNN trained on the CPU were recorded. The convolution, pooling, and three output layers were created for the FUIR-CNN model and connected. Then, applications were produced to determine the number display format to be used in the study. After the number display format to be used was determined, the unroll technique was applied to the loops to perform parallel operations in the convolution and pooling layers. The model developed in C++ was verified at the C level; thus, by verifying the developed model at the function level, time-consuming verifications with conventional hardware definition languages were completed easily and quickly. The model verified at the C level was synthesized and converted into an equivalent RTL circuit. After the designs were verified at the RTL level, they were converted to IP packages. Using IP packets, a connection was established between the mapping and the hardware created with the existing I/O pins.

#### 3.2.1. Data Types and Bit-Widths for the Tradeoff between Regression Accuracy, Latency, and Hardware Resource Usage

As number display formats, 32-bit single-precision floating-point number representation (32-bit FP) and fixed-point representation were used. For fixed-point representation, representations of different lengths (32-bits, 28-bits, 20-bits, 18-bits, and 16-bits) were tried, and their effects on resource usage, latency, and regression performance were examined. In the test phase, 204 I_F_–I_M_ image pairs were used. These images were applied to both the software- and hardware-based CNN models, and the predicted values were compared. The hardware-based CNN’s regression performance was calculated by comparing the values obtained using the software-based CNN.

**Figure 12:**
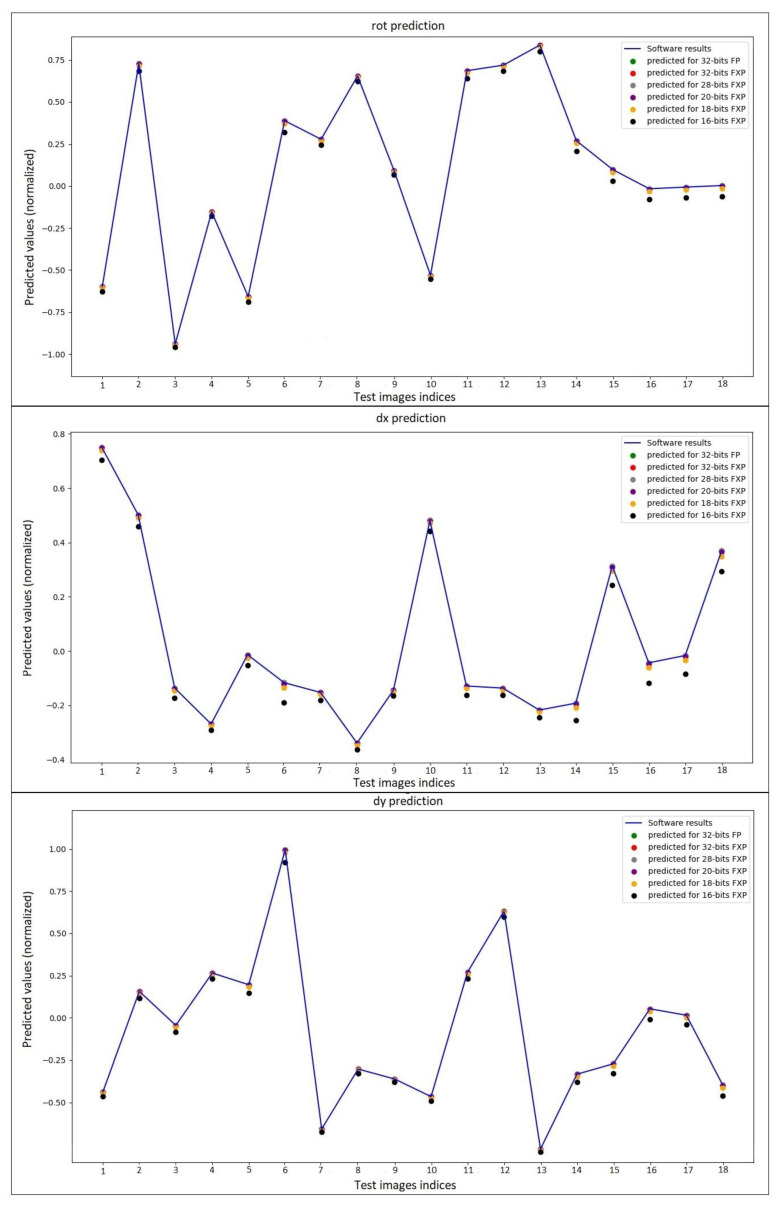
Results of 18 test US images for three outputs of the software and hardware models.

In Figure 12, the predictions of the models created using software-based CNN and different number representations for the 18 test images are shown in the graph. Since the ground-truth values used during the training of the network are normalized and given to the network, the output of the network also has normalized values between [−1,1]. Figure 12 shows 18 image indices on the horizontal axis and predicted normalized values on the vertical axis, along with software-based and hardware-based estimated values for the rot parameter at the top, the dx parameter in the middle, and the dy parameter at the bottom. Except for the model in which 18-bit and 16-bit number lengths are used, all points appear to be on this predicted line. It can be observed that the predicted points do not separate directly.

The estimation performance of the network for the number representation format and bit length to be used for hardware-based CNN is listed in Table 2. When the prediction performances were examined according to different metrics, the difference between the values produced by the software-based CNN and the results produced by the models implemented using 32-bit FP, 32-bit FXP, and 28-bit FXP was very small for all outputs. The data type of representation that produced the closest value was 32-bit FP. It can be observed that the loss of precision increases as the number of bits used for the representation of numbers decreases. According to the MSE metric for the rot output between the 20-bit representation and the 18-bit representation, the sensitivity loss is 4 times, and for 16-bit, there is a difference of almost 16 times. For R-squared, a value of 0.99 would mean that the variance of the independent variable accounts for 99% of the variance of the dependent variable under study. The R-squared value was calculated as 0.9999 for all models except for the model in which 18-bit and 16-bit number lengths were used. According to the results, accuracy suffers significantly when switching from 32-bit FXP to 16-bit FXP.

**Figure 13:**
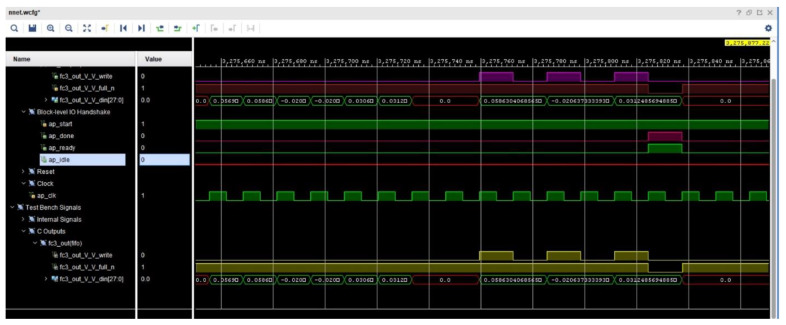
Simulated result trace file of CNN for 28-bit FXP.

Figure 13 shows the simulated result trace file of the CNN for the 28-bit FXP. A block-level I/O protocol was used in the design. There are four handshake signal ports in the protocol: ap_start, ap_idle, ap_done, and ap_ready. The design begins when the input signal ap_start reads as High. The signal ap_idle indicates that the design is idle. When ap_start goes to High, it immediately decreases. The ap_done port goes to High when operations are completed. The signal ap_ready goes to High to show that the model is ready to receive new input. The signal ap_start is always High; thus, the design does not enter the idle state, and the model starts to execute the next transaction immediately. If ap_start is High, ap_idle remains Low. The outputs of the network are expressed in FIFO units. Therefore, three information signals were used to determine the status of the FIFO: fc3_out_V_V_din, fc3_out_V_V_full_n, and fc3_out_V_V_write. When the model data are suitable for writing, the fc3_out_V_V_full_n information signal is checked. If the fc3_out_V_V_full_n signal is Low, it means that there is no free space in the FIFO, and a High signal for fc3_out_V_V_full_n means that data can be written to the FIFO. When the output data are valid, the output acknowledgment signal fc3_out_V_V_write is asserted. The FUIR-CNN designed in this study has three outputs; therefore, the fc3_out_V_V_write port was activated three times to write the three outputs. The information signal fc3_out_V_V_din shows the three output values written into the FIFO produced by the model.

In all the designs made for the number display, the clock frequency was determined to be 13 ns, and it was met. Table 3 displays the hardware resource usage and latency for different numbers of representations and FPGAs. In both FPGAs, the cost of 32-bit FP operations in terms of space and clock cycles is higher than that of 32-bit FXP operations. Using the FXP number display method, both the latency and hardware resources used were reduced. It can be observed that there is a difference between the resource usages, whereas the latency is the same for the 28-bit FXP and the 20-bit FXP. The applications were also compared using two different FPGA platforms: ZedBoard (xc7z020clg484-1) and Kintex UltraScale+ FPGA KCU116 (xcku5p-ffvb676-2-e). KCU116 has more resources, such as BRAM and DSP than ZedBoard does. The number of resources used increased with the number of optimizations. The resources available on the ZedBoard and clock frequency are insufficient for the proposed optimized design.

When performing a multiplication, the number of DSP48s that are required can vary depending on the format of the numbers being represented and the length of the data, as shown in Table 4. It shows the number of DSP48E sources used in the convolution layers for KCU116. The Conv1 and Conv2 layers used five DSP sources in convolution operations for 32-bit FP. To implement 32-bit × 32-bit FP multiplication, five DSP macros are required. When a 32-bit FXP is used, the number of DSP resources used decreases to 3. When the number of display lengths is selected as 20-, 18-, or 16-bits, only one DSP48 macro is required to implement multiplication.

#### 3.2.2. Computational Unit Optimizations

In an FPGA-based CNN implementation, parallel operations can be performed by unrolling loops. Therefore, the unroll method was used in the nested loops to accelerate operations in the calculation units. Before the loops were rolled, they were sequentially processed. Therefore, each iteration used specific hardware resources sequentially. This allows fewer resources to be used but creates a computational bottleneck. Applying the unroll method to the loops ensures that the transactions are performed in parallel at the determined factor, and separate resources are assigned for each parallel transaction. Thus, there will be a significant improvement in latency, and the number of hardware resources used will increase. The studies performed in the computational unit were compared using three models, as shown in Table 5.

Model 1 and Model 2 represented the CNN implemented using the 32-bit FP and 20-bit FXP number representation methods, respectively. There is no parallel processing in the layers of these two models. In other words, operations in the convolution, pooling, and FC layers are performed sequentially. Only the dataflow (pipelining) method was used between layers. The third model represents the model proposed in this study. In the proposed FUIR-CNN model, the fixed-point method was used as the data display method, and the data display length was chosen as 20 bits. In the FUIR-CNN model, the unroll method was used to perform parallel operations. In addition, the array-partitioning method was used to transmit data to parallel units.

**Figure 14:**
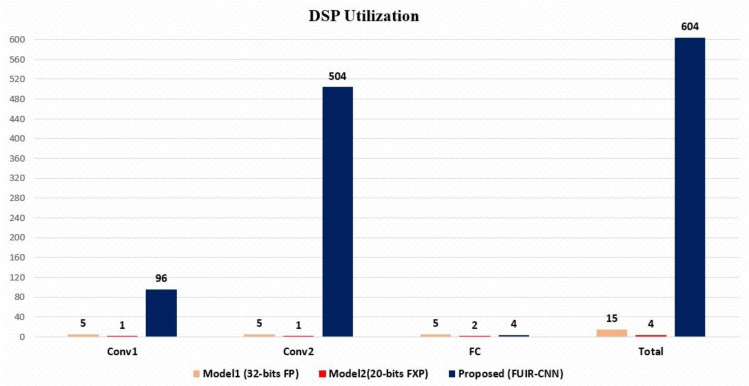
Number of resources used according to the layers.

Figure 14 shows the number of DSP resources used according to the layers for KCU116 FPGAs as a result of the implementation of the three models. When the number of resources used in the layers was examined, it was observed that the DSP resources were used intensively in the convolution layers. It was observed that 99% of the DSP resources used in the proposed model were used in the convolution layers. Only approximately 1% was used in the FC layer. This indicates that the processing load of the convolutional adders corresponds to the overall processing load of the network.

A total of 604 DSP hardware resources were used in the implementation of the proposed model. Of these, 600 were used in convolution layers; only 4 of them were used in the FC layer. This shows that more than 99% of the computational operations performed throughout the CNN network were performed in the convolution layers, so the convolution layers were computationally intensive. A total of 15 DSPs were used in Model 1, where the unroll optimization technique was not applied to the loops. A total of 4 DSPs were used in Model 2. The unroll method is not applied in this model; thus, all operations use the resources sequentially. In the proposed FUIR-CNN, when unroll is applied to the nested for loop, the number of DSP resources used increases to 604 because a resource is assigned to each process. In the Conv1 layer, 2 × 2 × 3 × 8 operations were performed in parallel, and 96 DSP resources were assigned for these processes. In the Conv2 layer, 2 × 2 × 8 × 16 operations were performed in parallel, and 504 DSP resources were assigned for these parallel operations. Four DSP sources were used in the FC layer, which was the output layer.

The resource utilization and latency of the three models are shown in Table 6. Model 1, which uses the high-precision number format, causes significant resource consumption when compared to Model 2. Using the floating-point number method causes more resource consumption and increases the latency compared to using fixed points. In the proposed method, the number of resources used increases as the processes are performed in parallel. Parallel processing in the proposed FUIR-CNN reduces latency by 27x when compared to Model 1 and 16x when compared to Model 2.

**Figure 15:**
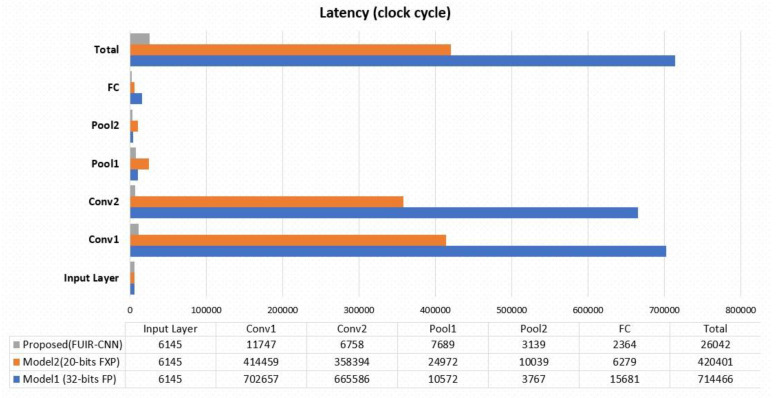
Latency for different layers.

Figure 15 shows the latency required to implement the three models. Latency is the number of clock cycles required for the function to calculate all of the output values. Since only the input image pair was stored in the input layer, there was no difference in latency between the models. However, the latency parameter differed due to the calculation processes in other layers. The clock cycles required to generate all outputs were 26,042, 420,401, and 714,466 for FUIR-CNN, Model 2, and Model 1, respectively. As a result of increasing the parallel processing units of the FPGA-based CNN application by applying unroll in nested loops, the latency value decreased significantly in each layer. In particular, a significant decrease was observed in the convolution layer. As shown in Figure 11, the number of clock cycles required in the first convolution layer of the proposed design decreased from 702,657 to 11,747 decreasing almost 35 times for Model 1. Similarly, in the second convolution layer, it decreased from 665,586 to 6758 decreasing almost 98 times for Model 1. Table 7 shows the execution times for the FPGA- and software-based test phases of the same CNN model.

The proposed FUIR-CNN required 26,042 clock cycles to complete all of the operations and write all of the outputs. A new image can be applied after 26,042 clock cycles. Regarding the completion time, the FUIR-CNN model was 27 and 16 times faster than Model 1 and Model 2, respectively, and 139 times faster than the software-based design.

Model 1 generated results approximately five times faster than the software-based work. Both models used 32-bit FP number representation. However, since the hardware-based Model 1 uses the dataflow optimization method, partial inter-layer parallelism is provided. This allowed it to produce results faster than the software-based model. Therefore, Model 1, whose layers worked concurrently, produced results five times faster than the CPU, which did not work concurrently. In Model 2, with the use of 20-bit FXP instead of 32-bit FP, the difference in speed with the software-based operation increased 8.6 times. With the application of unroll and pipeline optimization methods to Model 2, calculations in the network were performed in parallel. This increased the speed of the recommended hardware-based study by 139 times compared to the software-based study. The FUIR-CNN model achieved a peak performance of 7580 frames per second at a working frequency of 200 MHz.

**Figure 16:**
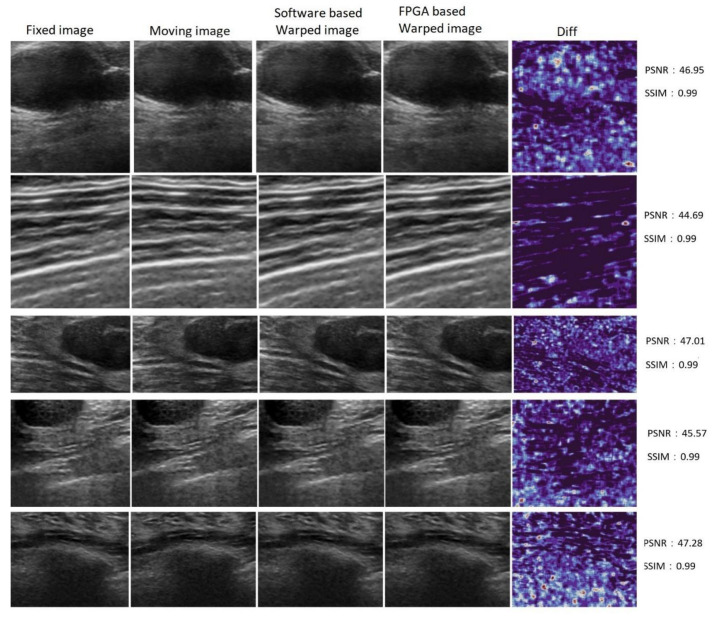
Fixed, moving, and warped images, and difference maps between software- and hardware-based warped images.

Figure 16 shows the warped images obtained after applying three estimated parameters to the moving image for the software and hardware CNNs and measuring the similarities between these warped images with SSIM and PSNR metrics. The images in the first and second columns demonstrate the I_F_ and I_M_, respectively. The images in the third and fourth columns show the I_WS_ and I_WH_ obtained from the software-based CNN and FUIR-CNN, respectively. The last column shows the difference between the two warped images. Also, SSIM and PSNR values between warped images are shown in Figure 12 for the five test images. According to the SSIM and PSNR metrics, the similarity across all of the sample images was calculated at 0.99 and 46, respectively, for 204 test images.

**Figure 17:**
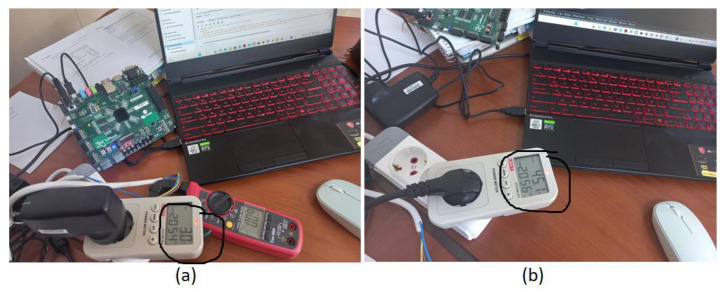
Power measurement of (**a**) the ZedBoard and (**b**) the laptop during the inference phase.

In addition to the negligible difference between the results produced by the software and those produced by the FPGA, less power was consumed by the FPGA, and the result was produced 139 times faster than that obtained with the software. On the KCU 116 FPGA, 200 MHz was achieved, but not on the ZedBoard for the proposed design. As the number of parallel operations increased, the number of DSP resources used also increased. In order to implement FUIR-CNN, 605 DSP hardware sources are needed, but ZedBoard has only 220 DSP sources. There are 1824 DSP sources in KCU116. Therefore, FUIR-CNN could not be implemented in real time on ZedBoard. Only Model 1 and Model 2 were successfully implemented on ZedBoard in real time. FUIR-CNN was successfully synthesized on KCU116 and IP packets were generated. In this study, while real-time power measurement was performed for ZedBoard, power measurement estimation was performed for the KCU using a Xilinx Power Estimator (XPE). According to the power estimate report produced by XPE after the successful implementation of the FUIR-CNN network, the sum of static and dynamic powers was calculated at 0.554 W for FUIR-CNN. The power measurement for the ZedBoard and laptop was performed using the power meter UT230B-EU. This device, which can measure power in the range of 0~3680 W, is used by plugging it into the power socket. Although the power consumption of the computer was calculated as 45 W on average in the registration process on the CPU during the extraction phase, that of the ZedBoard platform was calculated as 3 W, as shown in Figure 17.

Table 8 shows the comparison of CPU- or GPU-based medical image registration. As seen in Table 8, all of the DL-based studies produced results much faster than those using the traditional method. In this study, the input image size was smaller than in other studies. The FUIR-CNN proposed in this study produced much faster results than CPU- and GPU-based studies.

**Figure 18:**
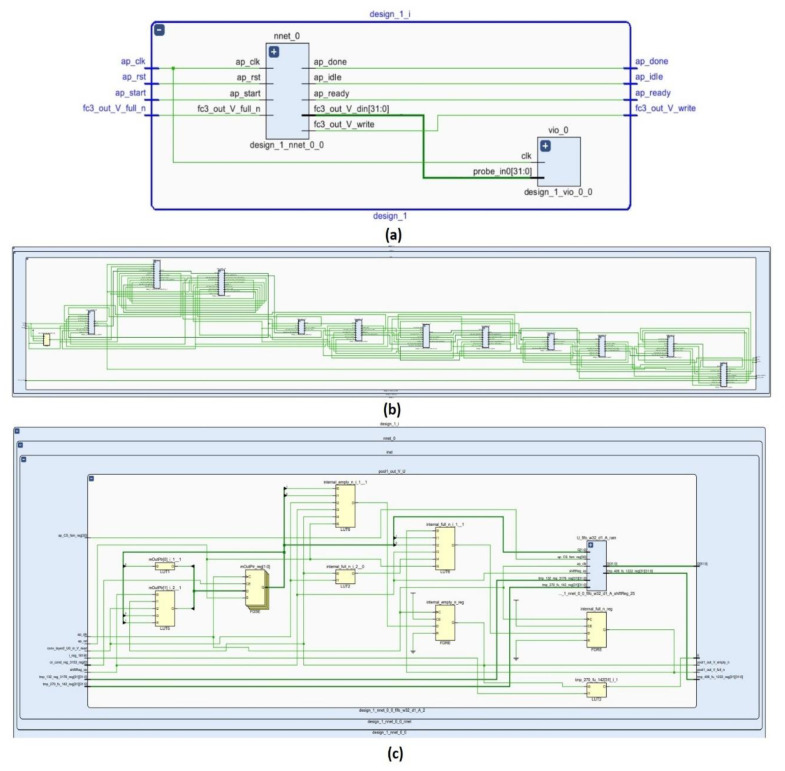
(**a**) A general schematic of the proposed hardware-based CNN network, (**b**) a schematic representation of all the layers and their interconnections, and (**c**) the pool1 layer and its connections.

Figure 18 shows (a) the general schematic of the proposed CNN, (b) a schematic representation of all layers and their interconnections, and (c) the pool1 layer and its connections. In order to show all of the values formed at the output of the design on the LEDs, the design was produced using the virtual input/output method because there were not sufficient LEDs in the FPGAs used. In Figure 14, a block diagram of the general structure of the CNN and the added virtual I/O block diagrams are shown. The Model 1 and Model 2 designs were successfully embedded in the ZedBoard in real time.

## 4. Discussion and Conclusions

It is vital to perform image registration accurately and quickly in image-guided surgical interventions. CNN-based IR methods have resulted in applications that respond faster than traditional iterative methods. CNN models are computationally intensive. Therefore, hardware structures capable of parallel processing, such as FPGAs, are required. This paper proposes an FPGA-based accelerated network FUIR-CNN model using the Vivado HLS tool for the estimation of US-to-US rigid image registration parameters at the inference state. The parameter-estimation performances of software-based and FPGA-based networks with the same hyperparameters and parameters were compared using the MSE, MAE, RMSE, and R-squared metrics. Only 0.0026, 0.0029, and 0.0025 losses occurred for rot, dx, and dy, respectively, according to the MAE metric, and 8.303 × 10^−6^, 1.053 × 10^−5^, and 7.772 × 10^−6^ losses, respectively, according to the MSE metric, in the prediction performance of the network. In addition, warped images created using the transform matrix produced by software- and hardware-based models as output were compared using SSIM and PSNR. The similarity between the warped images produced by the two models was calculated as 0.99 for the SSIM and 45 for the PSNR for 204 test images. In addition, in the hardware-based study, optimizations were performed on different number formats, different number display lengths, and nested loops, thus accelerating the regression network. In the proposed FUIR-CNN model, a parallel model is implemented by using dataflow, pipeline, unroll, and array-partitioning methods, reducing the latency. Considering the optimization methods used to perform the convolution operation in parallel, the proposed design produced results 139 times faster than the software model. Furthermore, in addition to their use in the medical field, the image registration process and CNN are employed in a variety of other fields. This demonstrates the broader applicability and impact potential of the proposed FPGA-accelerated CNN. There is still work to be done, even though the parameters needed for the US IR process in this study have been successfully estimated and performed faster than the CPU. It was observed that the hardware-based application provides faster results while retaining a negligible drop in regression performance compared to the software-based application and consumes less power at the same time. Moreover, it was observed that the resource usage, power consumption, and latency of the same models differed according to the FPGA platform utilized. In this study, the ZedBoard FPGA card was used and the number of DSP resources this card has is low; thus, the designed model cannot reach its maximum parallelism. Therefore, the proposed model was simulated using KCU 116. An important problem encountered in this study is that as the number of layers used increases, the number of errors due to the number of display methods employed increases. In future studies, the increase in error according to the number of layers will be modeled, and a more detailed study will be conducted. In addition, future research will examine other models that employ deep learning and machine-learning techniques besides CNN in terms of a variety of parameters, such as speed, power consumption, complexity, and hardware-resource utilization.

## Figures and Tables

**Table 1:** CNN hyperparameters determined for US-to-US IR.

Layers	Output Shape	Kernel Size	Kernel Number	Stride	Parameters
Input	(32, 32, 3)				
Conv2D	(31, 31, 8)	(2, 2)	8	1	104
MaxPooling2D	(15, 15, 8)	(2, 2)		2	0
Conv2D	(14, 14, 16)	(2, 2)	16	1	528
MaxPooling2D	(7, 7, 16)	(2, 2)		2	0
Flatten	784				0
Dropout	784				0
rot	1				784
dx	1				784
dy	1				784
Total parameters and trainable parameters					2984

**Table 2:** Regression performance of implementations for different evaluations.

	MAE			MSE			RMSE			R-Squared		
	rot	dx	dy	rot	dx	dy	rot	dx	dy	rot	dx	dy
**32-bits FP**	1.412 × 10^−6^	1.758 × 10^−6^	2.011 × 10^−6^	2.997 × 10^−12^	5.508 × 10^−12^	2.474 × 10^−6^	1.731 × 10^−6^	2.346 × 10^−6^	2.474 × 10^−6^	0.9999	0.9999	0.9999
**32-bits FXP**	1.555 × 10^−6^	1.599 × 10^−6^	2.366 × 10^−6^	3.370 × 10^−12^	4.086 × 10^−12^	7.311 × 10^−12^	1.835 × 10^−6^	2.021 × 10^−6^	2.703 × 10^−6^	0.9999	0.9999	0.9999
**28-bits FXP**	1.152 × 10^−5^	1.089 × 10^−5^	1.086 × 10^−5^	1.450 × 10^−10^	1.308 × 10^−10^	1.299 × 10^−10^	1.204 × 10^−5^	1.144 × 10^−5^	1.139 × 10^−5^	0.9999	0.9999	0.9999
**20-bits FXP**	0.0026	0.0029	0.0025	8.303 × 10^−6^	1.053 × 10^−5^	7.772 × 10^−6^	0.0021	0.0032	0.0027	0.9999	0.9998	0.9999
**18-bits FXP**	0.0106	0.0116	0.0099	0.0001	0.0001	0.0001	0.0114	0.0126	0.0106	0.9995	0.9982	0.9994
**16-bits FXP**	0.0425	0.0451	0.0399	0.0021	0.0024	0.0018	0.0452	0.0493	0.0434	0.9995	0.9982	0.9994

**Table 3:** Hardware resource usage and latency.

	ZEDBOARD (XC7Z020CLG484-1)					KINTEX KCU116 (XCKU5P-FFVB676-2-E)				
	BRAM18k	DSP48E	FFs	LUTs	Latency	BRAM18k	DSP48E	FFs	LUTs	Latency
32-BITS FP	18	15	33,069	12,833	1,006,610	16	15	32,555	10,941	714,466
32-BITS FXP	17	12	31,452	5926	512,657	14	12	30,982	5884	320,457
28-BITS FXP	14	12	27,472	5893	420,401	13	12	27,166	5878	320,457
20-BITS FXP	10	4	19,730	5491	420,401	10	4	19,534	5460	320,457
18-BITS FXP	10	4	17,795	5417	420,401	10	4	17,626	5386	320,457
16-BITS FXP	9	4	15,871	5367	420,401	8	4	15,718	5328	320,457

**Table 4:** DSP48 utilization for convolution layers.

	32-Bits FP	32-Bits FXP	28-Bits FXP	20-Bits FXP	18-Bits FXP	16-Bits FXP
Conv1	5	3	3	1	1	1
Conv2	5	3	3	1	1	1

**Table 5:** Models benchmarked for the computational unit.

	Data Represent Format	Lengths	Dataflow	Unrolling	Partitioning
Model1	Floating-point	32 bits	+	−	−
Model2	Fixed-point	20 bits	+	−	−
Proposed (FUIR-CNN)	Fixed-point	20 bits	+	+	+

**Table 6:** Resource utilization and latency of the three models.

	BRAM_18K	DSP48E	FF	LUT	Latency
Model 1	16	15	32,555	10,941	714,466
Model 2	10	4	19,677	5494	420,401
Proposed	74	604	43,301	38,735	26,042

**Table 7:** Latency comparison.

	Software	Model 1	Model 2	FUIR-CNN
Latency	0.018125 s	3.5723 ms	2.102 ms	0.13021 ms
Speedup	1x	5x	8.6x	139x
Images/second	55	280	476	7580

**Table 8:** Comparison to previous work based on medical image registration.

	[29]	[30]	[31]	[51]	[52]	[53]	This Work
Image size	128 × 128 × 128	512 × 512 × 323	64 × 64	512 × 512 × 150	256 × 256, 512 × 512, 400 × 400	512 × 512	32 × 32 × 3
Method	Supervised	Unsupervised	Unsupervised	Traditional method	Supervised	Unsupervised	Supervised
Transformation	Deformable	Deformable	Rigid	Deformable	Rigid	Deformable	Rigid
Modality	CT	CT/US	US	CT	CT	CT	US
Running time	0.55 s	0.7 s	0.1 s	58 min	0.1 s	1.67 s	0.13021 ms

## Data Availability

The datasets analyzed during the current study are available in the following repositories (http://splab.cz/en/download/databaze/ultrasound (accessed on 1 June 2023), https://scholar.cu.edu.eg/?q=afahmy/pages/dataset (accessed on 1 June 2023). http://www2.docm.mmu.ac.uk/STAFF/m.yap/dataset.php (accessed on 1 June 2023).
